# Report of the 47th annual conference and the golden jubilee year meeting of the Environmental Mutagen Society of India (EMSI) and international conference on environmental and molecular mutagenesis: genomic integrity and implication to human health, Tamil Nadu, India, January 29–31, 2025

**DOI:** 10.1186/s41021-025-00331-1

**Published:** 2025-04-25

**Authors:** Bani Bandana Ganguly

**Affiliations:** 1 MGM Center for Genetics Research and Diagnosis, MGM New Bombay Hospital, Vashi Sector 3, Navi Mumbai, 400703 India; 2 MGM Institute of Health Sciences, Navi Mumbai, 410209 India

**Keywords:** Environmental Mutagen Society of India (EMSI), The Japanese Environmental Mutagen and Genome Society (JEMS), UK Environmental Mutagen Society (UK-EMS), Mutagenicity and carcinogenicity

## Abstract

The 47th Annual Conference and the Golden Jubilee Year Meeting of the Environmental Mutagen Society of India (EMSI) and International Conference on Environmental and Molecular Mutagenesis: Genomic Integrity and Implication to Human Health was held at the Department of Biochemistry and Biotechnology, Annamalai University, Tamil Nadu, India, during January 29–31, 2025. Among the 18 international speakers, the former president of The Japanese Environmental Mutagen and Genome Society, the former and present presidents of UK Environmental Mutagen Societies (EMS) and the Office Bearers of the Indian EMS participated in the conference. The pre-conference workshop was held at the same venue one day before the main conference. Plenary and invited lecturers spoke about the assay systems, study parameters, biomarkers of disease onset, regulatory issues, and technological advancements in mutagenicity and carcinogenicity research. In brief: the effects of pesticides, heavy metals, nanoparticles, pharmaceutical impurities, UV-radiation, etc. on DNA damage and alterations in signalling and metabolic pathways were discussed. Discussion on errors in DNA-repair leading to disease-onset, remediation of genotoxicity with phytochemicals, identification of drug candidates, and progress in technological advancements such as error corrected Next Generation Sequencing (ecNGS) justified the theme of the Mutagen Societies. Altogether, 12 plenaries, 37 invited lectures, and general presentations, including 42 oral and 80 posters made the conference a grand success through lively interactive discussions. The organising team and EMSI expressed sincere thanks and gratitude to all the participants.

## Inauguration of the meeting

The 47th Annual Conference and the Golden Jubilee Year Meeting of the Environmental Mutagen Society of India (EMSI) and International Conference on Environmental and Molecular Mutagenesis: Genomic Integrity and Implication to Human Health was held at the Biochemistry and Biotechnology Department of Annamalai University, Tamil Nadu, India during January 29–31, 2025. At the same venue, a pre-conference workshop on Methods in Environmental Mutagenesis and Carcinogenesis was held on January 28, 2025. At the inauguration event, the Organising Secretary, Dr. N. Rajendra Prasad, welcomed the participants of the conference. Dr. Bani Bandana Ganguly, President of EMSI spoke about the history and Mission of EMSI and encouraged young researchers to become members of the society and pursue research on environmental health. She mentioned the affiliation of EMSI with the Asian Association of Environmental Mutagen Societies (AAEMS) and the International Association of Environmental Mutagenesis and Genomics Societies (IAEMGS), and the contribution of EMSI in India. EMSI was established in 1975 by a group of devoted and pioneering scientists, mainly from Bhabha Atomic Research Centre, Mumbai. She mentioned that she became a life member in 1986 when she attended the conference for the first time, and since then she attended EMSI conference almost every year as the annual conference of EMSI was a very attractive event to her.

She raised a concern about the reduced membership of EMSI over the years. Though there is a list of 450 members, many of them have deceased and many are not reachable due mainly to their non-responsiveness and absence in EMSI meetings. Contact details could not be updated for all life members. The Election Officer of the last election held in 2023 could connect only ~ 150 members. Keeping the global concern of environmental pollution in mind, young researchers have to be inspired to pursue careers in the field. Of course, technological advancements must be kept in place to monitor the environment and health. The load of toxicants is being augmented in the environment in parallel with industrial growth. Intoxicated flora and fauna are ultimately affecting human health. Hence, the problem is not restricted to any geographical boundary. EMSI shall contribute to public life by introducing and adopting research policies outlined by EMS societies and conducting outreach programs. Young researchers may be guided to lead future environmental research and develop policies to curb and mitigate genotoxic elements and their effects.

India is the most populous country in the world with diversified geography and cultures, distinctive lifestyles and religious customs, and so is the variation in environmental conditions and financial strength the people from different regions and strata face. Consequently, the effects of living conditions and occupational environments exert variable extent of health hazards at the backdrop of individual genetic makeup and lifestyles. This year, the theme of the conference was aptly decided as the implication of environmental mutagens and carcinogens to human health. The pre-conference workshop mainly discussed the theoretical and practical aspects of methodologies employed in the assessment of environmental and molecular mutagenesis for a batch size of ~ 80 participants. The faculties, including three international and four national experts, discussed the evolution of mutagenesis, research integrity, biomarkers of mutagenesis and carcinogenesis, chemical-induced carcinogenesis in experimental models, and existing and emerging methodologies for detection of the mutagens and carcinogens.

## Content of the conference

During January 29–31, 2025, overall attendance at the conference was good, especially for speakers of plenary lectures (PL) (100%). The noticeable feature of the conference was participation from several countries, including the USA, Austria, Japan, the United Kingdom, Singapore, Thailand, Italy, Malaysia, Uruguay, and Hungary. In addition, the present (Dr. Michael N. Routledge) and former (Dr. Anthony M. Lynch) Presidents of the UK Environmental Mutagen Society (UK-EMS), and the President, Secretary and Member of the Asian Association of Environmental Mutagen Society (AAEMS) and the Japanese Environmental Mutagen and Genome Society (Japan-EMGS) (Dr. Masamitsu Honma) were present. The present President, the immediate past and present Secretaries, and the immediate past and present Vice-Presidents of EMSI were also present at the conference. Several Editors of Mutation Research journals and distinguished scientists from several reputed institutions from India and abroad (Dr. Takayoshi Suzuki, NIHS, Japan; Dr. Roberta Bulla, University of Trieste, Italy; Dr. Ruth Morse, UK; to name a few; details are available in the conference proceeding) delivered lectures on different aspects of mutagenicity and carcinogenicity assessments and effects on human health [1].

The pre-conference workshop and the deliberations of plenary (PL) and invited (IL) lectures brought out the key insights of technological advancements and OECD guidelines on environmental research. Participation from research institutions in several developed countries has truly presented the current picture of technological advancement and its application in detection of biomarkers of genotoxic effects. The deliberations in PLs and ILs matched with the conference theme. The discussion on assay systems, parameters and technologies for detection of impacts of gene-environment interaction on human health justified the mission of EMSI. Lectures covering identification of biomarkers of exposure assessment/health effects, monitoring pharmaceutical impurities, finding inhibitors of non-homologous DNA end-joining, application of error corrected next generation sequencing (ecNGS) and the enhanced Ames test in mutagenicity assay, dietary interventions for mitigating telomere dysfunction and DNA damage, expression of chromosomal rearrangements in hematopoietic neoplasia and heritable disorders, genotoxicity of chemotherapy and radiation effects, the role of epigenomics and exposomics, regulatory issues related to genotoxicity assessment of medical devices, multi-omics approach for understanding pathways of disease-development and so on demonstrated a broad spectrum of advancement in mutagenicity and carcinogenicity research [1]. The Executive Committee Meeting and the Annual General Body Meeting of EMSI were also held at the same venue during the conference. It was proposed to change the existing EMSI name to The Environmental Mutagen and Genomics Society of India (EMGSI) since genomic studies are gaining importance for understanding the impact of mutagens and carcinogens on human health. Amendment of Bylaws may be required for the renaming of the society.

There were two parallel sessions covering 12 PLs and 37 ILs, and 42 oral presentations (OP) distributed on two different floors. In addition, a total of 80 posters have comprehensively displayed a wide array of technologies, biomarkers, assay systems, study parameters, and pathways and signalling systems toward understanding the environmental composition and mechanisms of action on human health by young researchers. Scientific talks on environmental health, including the effects of pesticides, plastics, particulate matter (PM2.5), drug ingredients, nanoparticles, environmental vector-borne diseases and host-vector interactions, radiation, etc. on one hand and bioremediation of the toxicity by microorganisms, plant extracts, and gene editing on the other were attractive. There were several lectures on DNA damage and repair mechanisms with reference to alterations in biological mechanisms of disease onset and progression such as metabolic pathways, signaling systems in tumour microenvironment, immune-modulation, epigenetic alterations, and their impact on human genome resulting in communicable and non-communicable diseases. Alterations in genetic signalling in different types of cancer cells and autoimmune disorders, and interaction with treatment modalities created a very interactive discussion in the conference. The amelioration of the alterations by synergistic interaction with phyto-extracts, immune suppressors/modulators, identification of biological molecules and bio-mechanism involved in disease onset, and identification of small molecule inhibitors and/or genetic manipulation made the conference a successful event.

Dr. Suzuki and Dr. Lynch demonstrated the application and regulatory issues of ecNGS. Dr. Lynch further elaborated on the precision of the enhanced Ames test in mutagenicity assay. Indeed, ecNGS enables the detection of chemical-specific non-biased signature mutations via specialized tagging strategies and bioinformatics processing, and can be applied for direct detection of low-frequency mutations in all models, including humans both in vitro and in vivo and in any tissue or genome. Quantitative and qualitative structure-activity relationship approaches have been explained by Dr. Honma in genotoxic research of chemical exposure. Assessment of the carcinogenic potential of drug impurities such as nitrosamines (*N*-nitrosodimethylamine (NDMA), and *N*-nitrosodiethylamine (NDEA)) was discussed by Dr. Lynch and Dr. Honma. In line, Dr. Lynch stated that OECD has approved a Standard Project Submission Form (SPSF; Project 4.175) in April, 2024 to develop a detailed review paper to subsequently amend the relevant OECD guidelines and to incorporate ecNGS as a tool for evaluation of mutagenicity in vivo and in vitro. Dr. Routledge discussed the Childhood Acute Illness and Nutrition (CHAIN) network study conducted on acutely ill children hospitalized in four African and two South Asian countries [2]. The children were exposed to food-borne aflatoxins. The targeted metabolomics and KEGG enrichment analysis revealed perturbation in 14 metabolic pathways suggesting an increased risk of liver-impaired growth, compromised cell-mediated immune system, chronic hepatomegaly and cancer. It was demonstrated that chronic aflatoxin exposure leads to aflatoxin-albumin adduct formation, which may lead to mortality in non-wasted children through altered intestinal function and disturbed glutathione metabolism resulting in inflammation, liver disease, and oxidative stress.

Epigenomic research on exposure assessment of pesticides, endocrine disruptors, etc. and their effects on human health, especially for risk assessment of cancer, diabetes, and birth defects is rapidly evolving. The methodologies ranging from genomics, transcriptomics, metabolomics and epigenomics create powerful multidimensional framework for understanding the effects of and susceptibility to biomarkers. ‘Exposomes’ represent a new frontier in environmental research to assess multiple factors simultaneously, including nutritional, behavioural and global environmental. Dr. Nina T. Holland (UC Berkeley, USA) talked about the influence of prenatal exposure to phthalates, a group of endocrine-disrupting chemicals consumed through the use of plastics and personal care products, on a wide range of developmental and health outcomes in children, including epigenetic aging during specific stages of child development. The hypermethylation study revealed an association between multiple CpG sites and phthalate metabolite concentrations, and differential methylation response (DMR) in genes associated with inflammatory response (IRAK4 and ESM1), cancer (BRCA1 and LASP1), endocrine function (CNPY1), and male fertility (IFT140, TESC, and PRDM8) suggesting differential DNA methylation causing adverse effects in newborns with prenatal phthalate exposure [3].

Whole genome bisulfite sequencing study revealed significant DNA damage and epigenetic changes (hyper- (1151), hypo- (309) and both (171) hyper- and hypo-methylation), in differentially methylated genes in normal human liver cells (WRL68) exposed to varying concentrations of chlorpyrifos (CPF; an organophosphate pesticide) [4]. CPF-exposure disrupted cellular integrity and biological pathways involving DNA repair genes, oxidative stress response, DNA methyltransferases and cell cycle markers, epithelial to mesenchymal transition markers and apoptosis, which collectively indicated potential risk of neoplastic transformation in liver cells.

The inflammation in mesothelial cells caused by environmental exposure to asbestos is managed by the complement system and macrophages, where C1q plays the first line of defence by recognizing the signals of danger and clearing of pathogens, and apoptotic and necrotic cells. C1q is abundantly expressed in the tumor microenvironment promoting the activities of solid tumor. Dr. Roberta Bulla showed immunosuppressive properties of C1q alongside its function for triggering the complement cascade and in the opsonisation process of phagocytosis and clearance of apoptotic cells. Exposure to asbestos fibres leads to downregulation of C1q by macrophages affecting clearance of apoptotic cells. Asbestos fibres can induce a switch of resting macrophages to an M1-like phenotypic profile, while expression of their characteristic markers such as CD206 and IL10 decreased in M2-polarised cells. Thus, C1q also induces polarisation of macrophages in an anti-inflammatory phenotype [5]. The study indicated that C1q marker can be used for identification of therapeutic targets in translational research. Dr. Sathees Raghavan (IISc, India) added how small molecule inhibitors such as SCR7, SCR116 and SCR132 have gained attraction as potent non-homologous end-joining (NHEJ) inhibitors, which can accumulate unrepaired double-strand breaks (DSBs) in cancer cells leading to tumor regression in experimental mice models. The latter two compounds significantly reduced tumor progression in two mouse allograft models by triggering G_2_-M arrest and apoptosis [6]. The compounds reduced tumor progression and increased survival with no significant toxicity but increase in bioavailability suggesting its efficacy in therapeutic modalities of radio-and chemo-resistant cancer.

In this line, TGF-βR inhibitor SB431542 decreased the generation of T-regulatory cells and reduced the tumor burden in mice suggesting SB431542 as an epigenetic drug candidate. Also, a possible epigenetic therapy before radiotherapy in HPV-associated cervical cancer with histone deacetylase inhibitors (HDACi; valproic acid) and tobastatin A (HDAC6i) was estimated by MN- and COMET assay in human keratinocyte derived cell line (HaCaT cells) and that showed overexpression of E6 and E7oncoproteins. In non-small cell lung cancer, epigenetic modulation of T-helper cell differentiation indicated possible therapeutic intervention by targeting CTCF-associated pathways to bloster immune response. Nevertheless, heterogeneous pattern of HLA data in the Indian population was discussed to be attributed to the evolutionary pressure due to microbial load across various geographical regions, where population-specific HLA alleles may help in donor selection. Myricetin flavonoid significantly inhibited the interaction between IL-21 and IL-21R and suppressed JAK/STAT signalling and the downstream transcription factor Bcl-6, and reduced T follicular helper cell differentiation in Rheumatoid arthritis (RA), suggesting myricetin a therapeutic compound for RA.

Talks on antimutagenic and/or anticarcinogenic potential of a wide range of plant extracts such as: fenugreek seed powder nullified aluminium chloride-induced features of Alzheimer disease via Akt/GSK3β signalling; preventive effect of withanone on doxorubicin-mediated oxidative stress, apoptosis and fibrosis in cardiac tissue; protection of UV-induced skin inflammation and aging by flavonoids (epigenin and niringin), nobiletin, caffeic acid, ferulic acid, α-pinene through inhibitory action on inflammatory proteins (TNF-α, IL- 6, COX- 2) and NF-kB, STAT- 3, PTEN and PI3 K/Akt pathways; anti-oxidative and anti-inflammatory effects of theaflavin from black tea and curcumin on neuro-protection and neurodegeneration; synergistic impact of plant extract and antibiotics on *E. coli* and *S. aureus* to modulate the resistance to antibiotics; protection of mitochondrial integrity by cardioprotective antioxidant action of phloretin, a potent antioxidant flavonoid from apple peel extract, in arsenic trioxide-induced oxidative stress in H9c2 cells; significant amoebicidal and antiadhesion potential observed with medicinal plant extracts on *Acanthamoeba sp.* assessed by electron microscopy, molecular docking and dynamic simulation; and so on were interesting.

Melanin is known to protect skin from natural UV-induced DNA damage; however, intermediates of eumelanin are highly reactive quinones that are potentially genotoxic. Polk-mediated replication stress response to melanin-induced DNA damage showed the crucial role of translesion polymerase Polk as a safeguard against genome instability and to maintain genome integrity. The study emphasized the dual role of melanin in protecting and damaging melanocytes, and (eu)-melanogenesis’s link between tanning response and mutagenesis during pigment production [7]. In line, an association of severe inflammation and poorly differentiated hyper-proliferative keratinocytes with psoriasis was elucidated by understanding immunogenetics of adipokine genes among South Indian patients. Several biomarkers were discussed for assessing the environmental genotoxicity and patho-mechanism of disease onset. Abnormal cholesterol and PI3K/Akt.mTOR status was studied as predictive markers to screen high-grade serous ovarian cancers.

The economic burden on assessment of occupational health hazards was discussed with a model on agricultural workforce. Similar constraint on research funding leads to employment of conventional in vivo and in vitro techniques for evaluation of genotoxicity of accidental, environmental and occupational exposures to human health. The screening of immediate post-disaster and surveillance study of methyl isocyanate (MIC) exposure after 30 years on differentially exposed survivors did receive critical review comments on use of conventional G-banding, though detected significant information [8]. To support the use of this technique, vast data extracted from peripheral blood or bone marrow was presented. Nevertheless, the data corroborated with the genesis of genomic abnormalities and origin of chromosomal rearrangements and/or oncoproteins in relevance to exposure to environmental hazards, lifestyle and socio-demographic stressors was attractive. Thus, besides ecNGS and multiomics application, chromosome abnormalities covered a niche area in the conference, which was reflected in presentations covering conventional G-banding studies of chromosomal damage and rearrangements by Dr. Ganguly, FISH (fluorescence in situ hybridization) study of telomere dysfunction by Dr. Hande (NUS, Singapore), the importance of micronucleus assay by Dr. Knasmüller (Medical University of Vienna, Austria), and comet assay by several speakers. Nevertheless, OECD guidelines have established protocols for in vitro and in vivo genotoxicity assays. Dr. Knasmüller explained that micronucleus indicates DNA damage in buccal epithelial cells as a significant association with tobacco chewing in particular, and exposure to heavy metals and many more xenobiotics in general. Dr. Hande showed an intricate relationship between dietary interventions and telomere dynamics to understand the mechanistic effects of dietary restriction on telomere integrity and DNA repair. Prophylactic intermittent fasting extended lifespan by rendering genome stability, reducing spontaneous chromosomal damage and expression levels of protein involved in DNA damage response and telomere attrition in mice. Dietary calorie restriction may establish unprecedented health benefits and protection from age-related pathogenesis.

## Young talents

Three Awards were conferred for oral and three for poster presentations from Mutation Research journal. Awards were also given in the name of ‘Annamalai University Award’ to oral and poster presentations. Congratulations to all the awardees! Dr. Prakash Hande, one of the editors of the Mutation Research journal announced a special issue of the journal on Genetic Toxicology in India, and explained the guidelines for submission. It was quite satisfying to see that there will be a special issue of the journal on toxicology research in the Indian context of genotoxicity studies.

## Talent beyond science

A cultural program was hosted by the EMSI- 2025 organising committee to display the rich cultural history of Indian music. The students of the Music College, Annamalai University presented a mesmerizing fusion of beats and tunes of Indian music on one hand, and the rhythmic movement of limbs with facial expressions linking to dance patterns of different Indian states on the other hand by boys and girls respectively gave a musical treat to the conference participants gathered from different parts of the globe. It was truly no less than a ‘million dollar’ concert.

## Conclusion and perspectives

Indeed, organising a 4-day academic event requires a lot of sincere planning for a smooth execution. The academic side of the conference was very well managed by the organising team. However, there may be some unintentional gaps and deficiencies, which were handled carefully. In brief, the participation of senior scientists and young researchers from different countries made EMSI- 2025 a successful academic event. The organising team of EMSI-2025 deserves a big CONGRATULATION. The deliberations in the plenary and invited lectures highlighted the importance of appropriate methodologies, assay systems, genotoxic markers and handling of confounders, which may lead to a better understanding of the biological mechanism of disease onset and remediation of the effects by using phytochemicals or identification of small molecule inhibitors. Six young researchers won ‘Mutation Research’ award at this conference, which will motivate more young scientists to pursue career in environmental research. We hope EMSI will make the future meetings attractive with more participation and new members by taking care of the scientific and social part of the conference.

Employment of advanced technologies in genotoxic assessment, both in terms of facility and feasibility plays a pivotal role, but it requires financial support. The environmental research in developing countries has yet to adopt ecNGS or multiomics for assessment of exposure or disease-onset. It is thus very important to define policies on collaboration with the global platform of environmental research. Therefore, a dedicated session on possible collaborative research shall be defined and integrated into future meetings of mutagen societies, including EMSI, because the admixture of the atmospheric environment is shared by the global population ultimately. In addition, research can follow harmonised protocols and technicalities in assessments of chemical and/or radiation exposure. Moreover, the participation of pharmaceutical industries and policymakers may ease the collaborative approach of the assessment of environmental mutagenesis and carcinogenesis.

## Data Availability

No datasets were generated or analysed during the current study.

## Abbreviations

EMSI: Environmental Mutagen Society of India
JEMS: The Japanese Environmental Mutagen and Genome Society
UK-EMS: UK Environmental Mutagen Society
